# Corneal and Ocular Surface Contributions From Mexico: A Bibliometric Analysis From 1913 to 2022

**DOI:** 10.7759/cureus.66965

**Published:** 2024-08-15

**Authors:** David Jimenez-Collado, Braulio Hernán Velasco-Sepúlveda, Ángel Lee, Guillermo Raul Vera-Duarte, Enrique O Graue-Hernandez, Alejandro Navas

**Affiliations:** 1 Ophthalmology, Institute of Ophthalmology "Conde de Valenciana", Mexico City, MEX; 2 Cornea, Institute of Ophthalmology "Conde de Valenciana", Mexico City, MEX; 3 Neurological Endovascular Therapy, National Instituto of Neurology and Neurosurgery "Manuel Velasco Suarez", Mexico City, MEX

**Keywords:** research, publication productivity, ophthalmology, bibliometrix, ocular surface, bibliometric analysis

## Abstract

Objective: This study aimed to investigate all recorded corneal and ocular surface research by Mexican authors.

Methods: The output data was extracted from SCOPUS to account for all publications regarding the corneal or ocular surface by Mexican authors. Data screening, extraction, and critical revision were performed by two of the authors to avoid duplication and ensure the authenticity of all papers. Performance analysis, science mapping, and network metrics were employed to retrieve trends in publication.

Results: A total of 1,091 indexed journal documents by 3965 authors were retrieved, covering the period the period from 1919 to 2022. In performance analysis, the document types included 881 articles, 20 book chapters, 17 conference papers, three editorials, 37 letters to the editor, nine notes, and 123 reviews. A total of 3,965 contributing authors made 6,081 author appearances. In terms of total citations per country, Mexican authors received a total of 7,087 citations, with an average article citation of 8.76 per author.

Conclusion: This bibliometric analysis highlights impactful research contributions to corneal and ocular surface research from Mexican authors, identifies influential authors and institutions, and also emphasizes the need for increased interaction in the international arena.

## Introduction

Research is undoubtedly important to a country’s scientific development and progress. Biomedical research projects usually lead to publications in serial literature. Original articles allow investigators to present their observations, and the publication of that project allows the information to be shared with the scientific community. Furthermore, publications are often used to measure the success of research work performed by hospitals and institutions. In recent years, there has been growing interest in developing scientific indicators capable of facilitating the analysis of the results of research activities [1]. One way of doing so is to perform a bibliometric analysis, which can be regarded as a map of medical research and a study tool using quantitative indicators to understand productivity with characteristics and factors for a greater impact [2,3]. The determination of a citation hierarchy list in one specialty of the medical field, formed by numerous journals that are specific to one specialty, allows the discovery of influential authors and institutions as well as opportunity areas.

Work in ophthalmology has grown substantially in the last decades, and research regarding corneal and ocular surface topics has had a special and particular interest due to new modalities of surgery, the uprise of new technology, and cellular biology advances. Previous bibliometric analysis has been performed regarding ocular surface topics such as dry eye disease [2], and others have reviewed Mexican contributions on another topic [3,4]. However, no study has been published that addresses the matter of the scientific contribution of a whole country to one specific area of vision science knowledge. This paper will set the pace for the analysis of Mexican authors' contributions to ophthalmology, such as the most studied areas, factors for the impact of publications, characteristics of production, and most productive authors/institutions [3,4]. This study aimed to investigate all recorded corneal and ocular surface research by Mexican authors.

## Materials and methods

The output data was extracted from SCOPUS on December 22, 2022, by using the following query: ALL ( ( ( ( "cornea/abnormalities" ) OR "cornea/analysis" OR "cornea/anatomy and histology" OR "cornea/blood supply" OR "cornea/chemistry" OR "cornea/cytology" OR "cornea/diagnosis" OR "cornea/diagnostic imaging" OR "cornea/drug effects" OR "cornea/embryology" OR "cornea/enzymology" OR "cornea/etiology" OR "cornea/growth and development" OR "cornea/immunology" OR "cornea/injuries" OR "cornea/innervation" OR "cornea/isolation and purification" OR "cornea/metabolism" OR "cornea/microbiology" OR "cornea/parasitology" OR "cornea/pathology" OR "cornea/pharmacology" OR "cornea/physiology" OR "cornea/physiopathology" OR "cornea/radiation effects" OR "cornea/surgery" OR "cornea/therapy" OR "cornea/transplantation" OR "cornea/ultrastructure" OR "cornea/virology" ) OR "Cornea" ) ) AND ( LIMIT-TO ( AFFILCOUNTRY , "Mexico" ) ) AND ( LIMIT-TO ( SUBJAREA , "MEDI" ) ).

No language or year of publication limitation was placed on the articles. Information regarding title, authors, journal of publication, institutional affiliations, year, abstract, and keywords was collected. Data screening, extraction, and critical revision were performed by two of the authors (DJC and BVS) in order to avoid duplication and ensure the authenticity of all papers. Studies were only limited in the query to medicine-related articles. As this study involved the analysis of existing literature without any intervention with human subjects, ethics committee approval was not required.

For a more comprehensive analysis, we recorded variables like temporal profile, research subject, language of publication, type of research, journal of publication, level of evidence, geographic distribution of research output, international collaborations (defined as an article with at least one author having a non-Mexican institutional affiliation, regardless of their nationality), corresponding authors and institutions, and number of citations.

Regarding analysis techniques, performance analysis was performed to rate scientific achievements from different research constituents, science mapping was realized to examine interactions and collaborations among research institutions, and network metrics were used to provide greater clarity of centrality measures [5].

All data was entered into R version 4.0.2 (The R Foundation for Statistical Computing, Vienna, Austria), and the “bibliometrix” package was used for data analyses and visual networks [6]. Descriptive statistics are expressed with frequencies, percentages, or the mean plus standard deviation, where appropriate. The impact of scientific output was determined by the ratio of citations to publications (c/p) [3]. The VOSViewer software version 1.6.18 (Centre for Science and Technology Studies, Leiden University, The Netherlands) was used to plot all web network analyses.

## Results

Performance analysis

**Figure 1:**
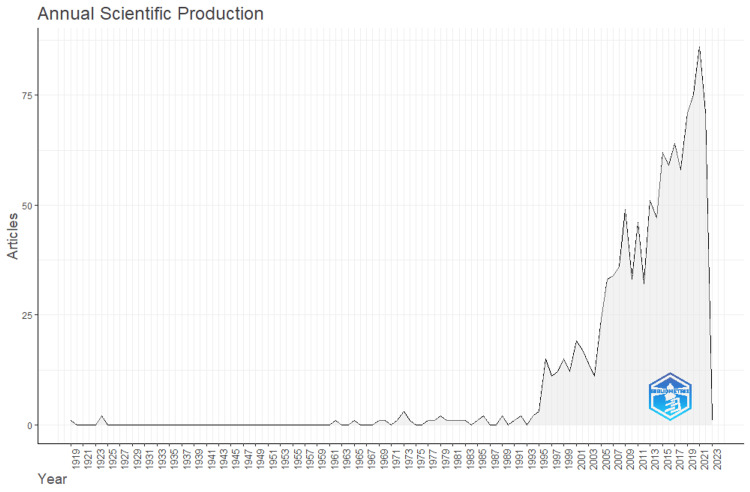
Density plot showing annual scientific production among Mexican authors

Our analysis retrieved 1,091 documents authored by 3,965 individuals, spanning the years from 1919 to 2022. The document types included 881 articles, 20 book chapters, 17 conference papers, three editorials, 37 letters to the editor, nine notes, 123 reviews (including literature, narrative, systematic/meta-analysis), and one short survey. Among these documents, 49 were single-authored, and 29.06% of the articles involved international co-authorships. The peak year of productivity was 2021. Figure 1 shows the annual scientific production.

From a total of 6,081 author appearances, they account for 3,965 different contributing authors. The collaboration index was calculated to be 3.63. This is obtained by dividing the number of authors (3,965) by the number of documents (1,091). The dominance factor was also calculated, and the highest-ranked author had a dominance factor of 0.36 [7].

Citation analysis by country

In terms of total citations per country, Mexican authors received a total of 7,087 citations in Mexican journals, with an average article citation of 8.76 per author. Authors from the following countries also cited Mexican authors in their research: the USA, Spain, the UK, the Netherlands, Singapore, Australia, Sweden, India, and Norway.

In the studied topics, the most relevant sources publishing articles from Mexican authors were (total number of papers in parenthesis): Revista Mexicana de Oftalmología (240), Cornea (48), Journal of Cataract and Refractive Surgery (34), Archivos de la Sociedad Española de Oftalmología (32), International Ophthalmology (25), Journal of Refractive Surgery (21), Investigative Ophthalmology and Visual Science (19), American Journal of Ophthalmology (18), British Journal of Ophthalmology (15), and Clinical Ophthalmology (15).

**Figure 2:**
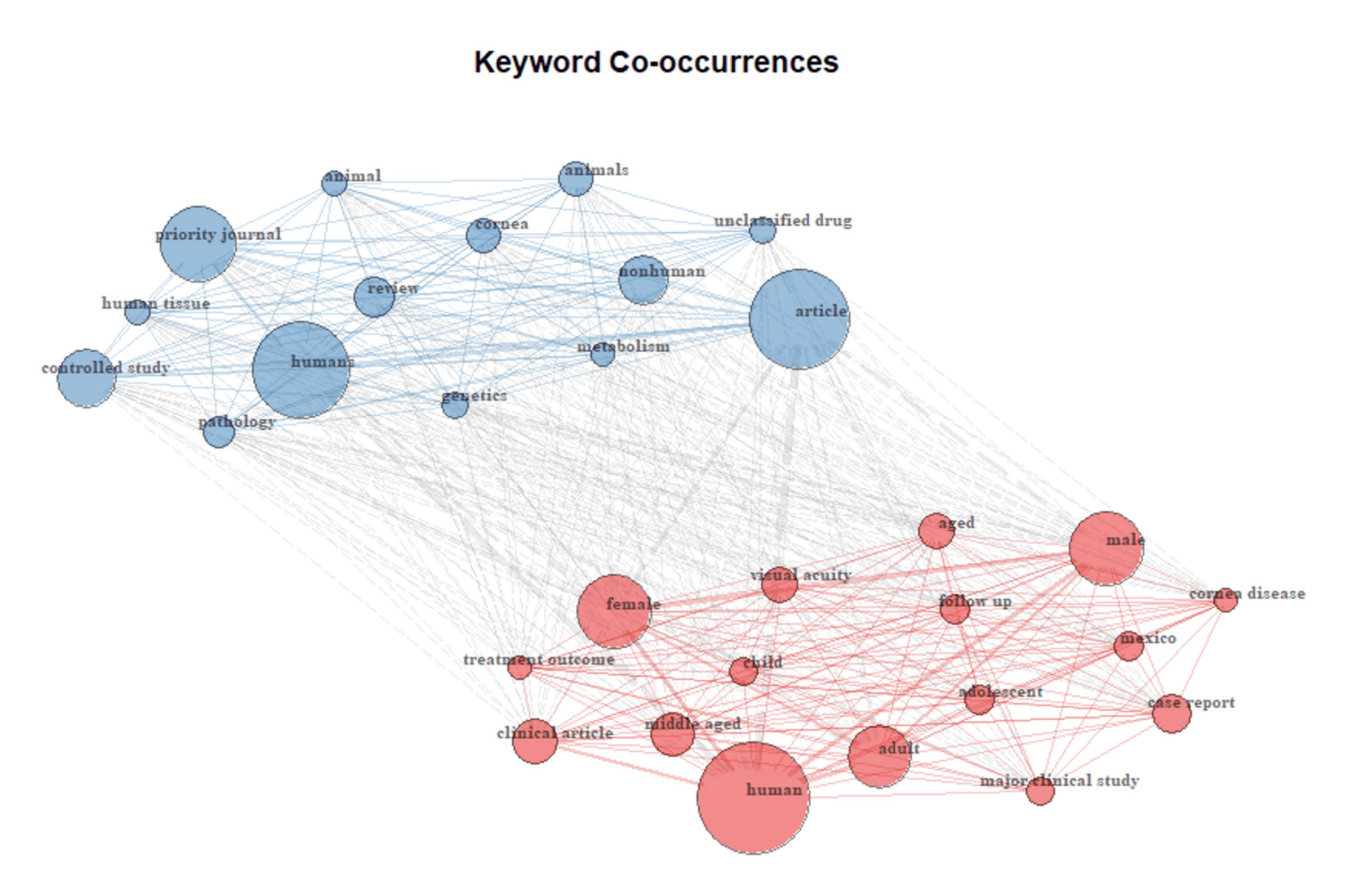
Network web plot showing main keyword co-occurrences

The most relevant keywords excluding sociodemographic terms (‘human', 'male‘, ‘female‘, ‘article‘, ‘humans‘, ‘adult', 'middle-aged‘, ‘aged‘, ‘visual acuity‘, and ‘priority journal‘) were as follows: ‘Mexico‘ cited by 43 articles (43), ‘cornea‘ (42), ‘keratoconus‘ (30), ‘keratitis‘ (26), ‘penetrating keratoplasty‘ (21), ‘glaucoma‘ (19), ‘LASIK‘ (18), ‘cataract‘ (16), and ‘dry eye‘ (16) (Figure 2).

Author productivity and citations

**Figure 3:**
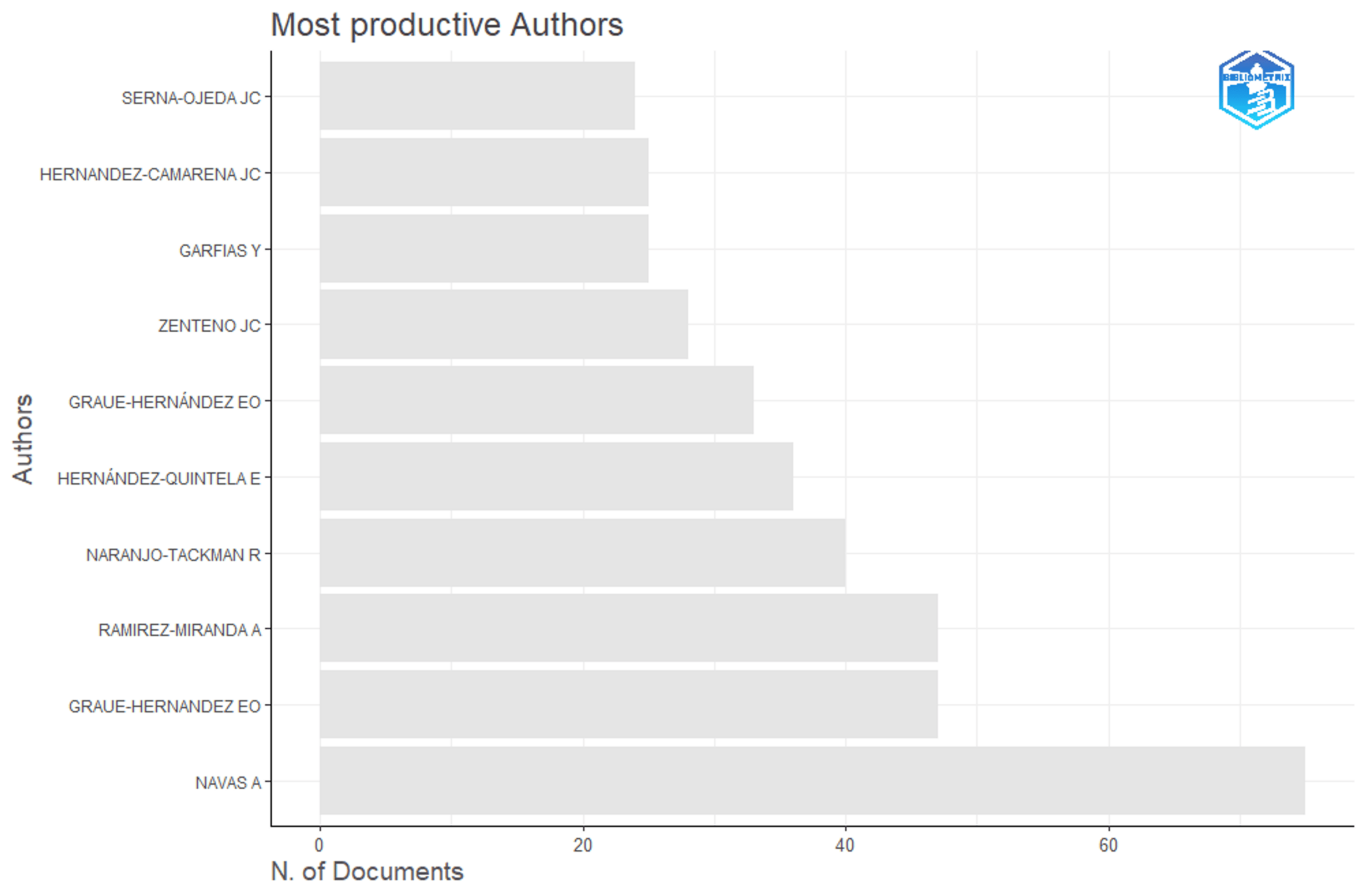
Horizontal bar graph showing productive Mexican authors

The authors with the highest productivity are as follows (name followed by total papers in parenthesis): Navas A (75), Graue-Hernandez EO (47), Ramirez-Miranda A (47), Naranjo-Tackman R (40), Hernández-Quintela E (36), Graue-Hernández EO (33), Zenteno JC (28), Garfias Y (25), Hernandez-Camarena JC (25), and Serna-Ojeda JC (24). Graue-Hernandez EO and Graue-Hernández EO are the same author but with a difference in the accent in their last name (Figure 3).

**Figure 4:**
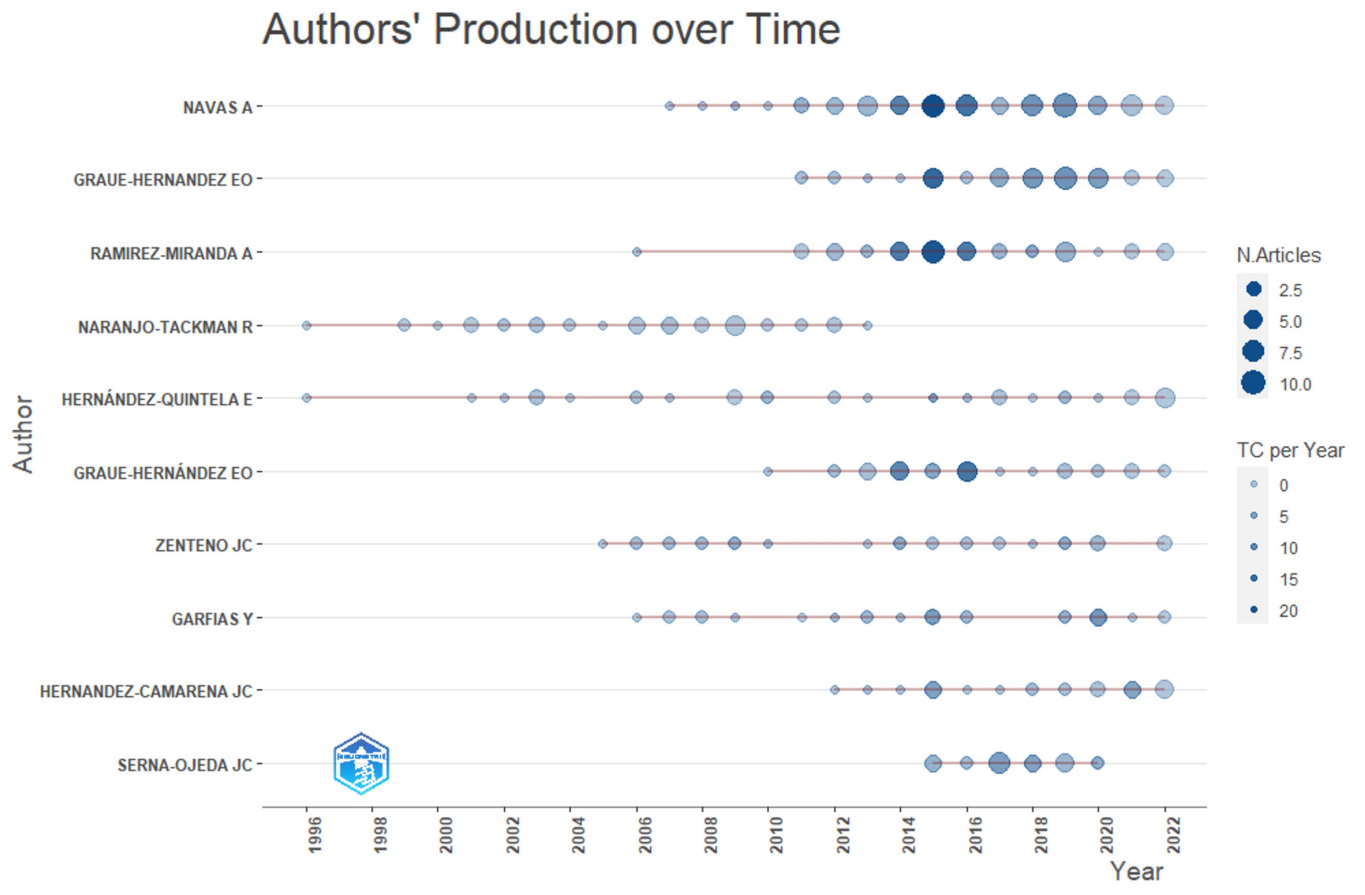
Horizontal lollipop plot demonstrating production over time among top Mexican authors from 1996 to 2022

The authors with the most local citations were (citations in parentheses): Navas A (20), Zenteno JC (16), Bautista-De Lucio VM (11), Hernandez-Camarena JC (11), Hernández-Quintela E (11), Ruiz-Lozano RE (11), Graue-Hernández EO (10), Graue-Hernandez EO (9), Garza-Leon M (9), and Lichtinger A (9) (Figures 2-3). The top 10 H-index-ranked authors are presented in Table 1 and Figure 4.

**Table 1:** Top 10 H-index-ranked authors

Author	Scopus ID	Documents	H-index	G-index
Graue-Hernandez EO	26647473200	47	11	17
Navas A	23012743000	75	15	26
Zenteno JC	7004122469	28	12	18
Ramírez-Miranda A	12804832700	47	13	23
Garfias Y	6507941116	25	12	15
Hernández-Quintela E	6603055501	36	7	14
Serna-Ojeda JC	55178461900	24	9	12
Hernández-Camarena JC	35519690900	25	6	12
Naranjo-Tackman R	6507147219	40	7	10
Graue-Hernández EO	26647473200	33	9	18

Science mapping

The most influential papers with at least one Mexican co-author and cited by international authors are summarized in Table 2.

**Table 2:** Papers most cited by international authors

	Title	Citations	Annual mean
1	EULAR recommendations for the management of Sjögren’s syndrome with topical and systemic therapies	189	63
2	Long-term results from an epiretinal prosthesis to restore sight to the blind	180	22.5
3	Regression and its mechanisms after laser in situ keratomileusis in moderate and high myopia	177	7.1
4	Keratitis, ichthyosis, and deafness (KID syndrome): review of the literature and proposal of a new terminology​​​​​​​	146	5.4
5	Central and peripheral corneal thickness measured with optical coherence tomography, Scheimpflug imaging, and ultrasound pachymetry in normal, keratoconus-suspect, and post–laser in situ keratomileusis eyes	130	9.3

“EULAR recommendations for the management of Sjögren’s syndrome with topical and systemic therapies“ [8] (189, 63) had the highest number of citations, followed by the subsequent publications: “Regression and its mechanisms after laser in situ keratomileusis in moderate and high myopia” [9] (177, 7.08), ”Keratitis, ichthyosis, and deafness (KID syndrome): review of the literature and proposal of a new terminology” [10] (146, 5.41), ”Central and peripheral corneal thickness measured with optical coherence tomography, Scheimpflug imaging, and ultrasound pachymetry in normal, keratoconus-suspect, and post-laser in situ keratomileusis eyes” [11] (130, 9.29). Table 3 shows the papers most cited by other Mexican authors.

**Table 3:** Papers most cited by Mexican authors

	Title	Citations
1	Keratoconus	7
2	Corneal reinnervation after photorefractive keratectomy and laser in situ keratomileusis: an in vivo study with a confocal videomicroscope	5
3	Confocal microscopy of corneal flap microfolds after LASIK	5
4	Molecular chaperones in the cytosol: from nascent chain to folded protein	4
5	Advances in the molecular genetics of corneal dystrophies	4
6	Keratoconus and related noninflammatory corneal thinning disorders	4
7	Is Acanthamoeba encephalitis an opportunistic infection?	4
8	Prevalence of and risk factors for dry eye syndrome	4
9	Reliability and validity of the ocular surface disease index	4
10	Ultrafast (femtosecond) laser refractive surgery	4

Below, we summarize the most cited papers by Mexican authors: "Keratoconus" [12] (7), "Corneal reinnervation after photorefractive keratectomy and laser in situ keratomileusis: an in vivo study with a confocal videomicroscope [13] (5), "Confocal microscopy of corneal flap microfolds after LASIK" [14] (5), and "Molecular chaperones in the cytosol: from nascent chain to folded protein [15] (4). All included articles are listed in Table 3.

**Figure 5:**
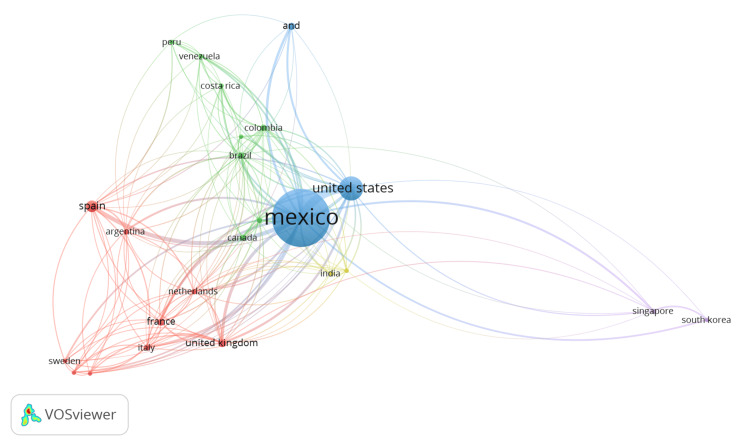
Network web plot on country collaboration

**Figure 6:**
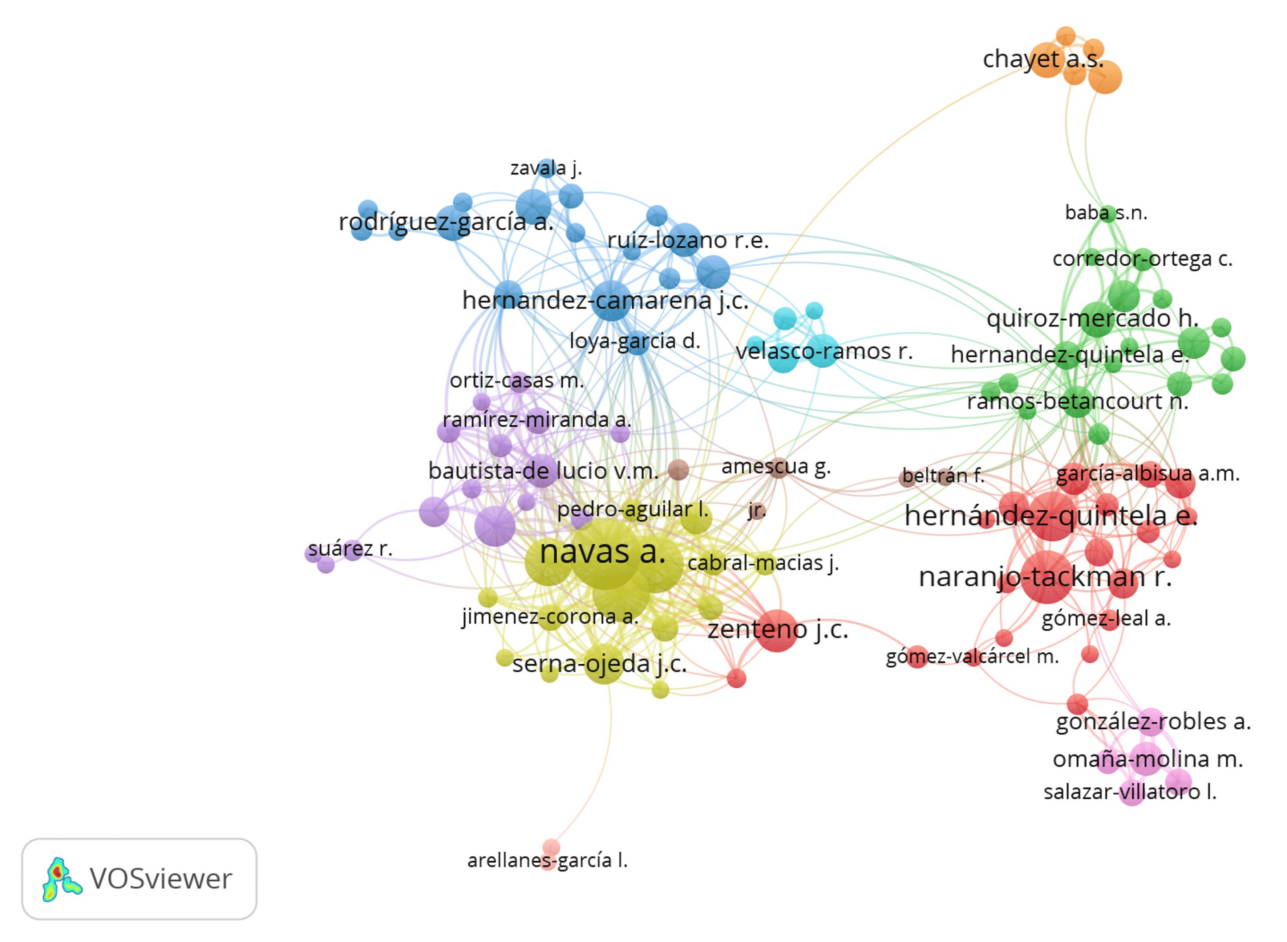
Network web plot analyzing collaboration between authors and author groups

Country collaboration (number of papers with collaborating countries) was strongest between Mexico and the following countries: the USA (80), Spain (17), Italy (7), Singapore (7), the UK (7), Canada (5), Brazil (3), India (3), and the Netherlands (3). The research web of these collaborations is shown in Figure 5. Network analysis between the different authors mentioned previously, showing the collaborations between the most productive groups in the country, is shown in Figure 6.

## Discussion

Bibliometric analysis is paramount to elucidating hotspots and knowledge gaps in research, but this method has been applied to Latin American vision science in only a few papers (Ragghianti 2006 [16], Ventura 2008 [17], and Galván 2018 [18]), and those targeting major countries in the region have only been centered in Brazil (Muccioli 2006 [19] and Lira 2013 [20]). Mexico is the largest Spanish-speaking country in the world, the 15th largest economy, and the only Latin American country belonging both to the Organisation for Economic Co-operation and Development and the G20, and bibliometric analysis has only recently been focused on its scientific production as a nation (Paceco Aispuro 2023 [21], Milla 2023 [22]). The Mexican literature in ophthalmology has never been analyzed, and particularly research on corneal diseases that represent 20% of Latin American papers (third subject after retina and strabismus) has never been studied in Latin America. Our paper will address this gap in the existing literature.

The corneal and ocular surfaces constitute one of the most crucial subspecialties in ophthalmological practice and research. In recent years, significant technological advancements have revolutionized the treatment and management of patients with corneal diseases [23,24]. These innovations underscore the need for new publications evaluating the safety and repeatability of novel surgical techniques and the application of emerging devices across diverse populations. Approximately 4.5 million people worldwide experience moderate to severe vision impairment due to corneal diseases, while an additional 101.2 million suffer from refractive errors such as myopia, hypermetropia, and astigmatism [25].

Consequently, investigating corneal pathologies and refractive surgery is of paramount importance. Analyzing the research contributions of a single country in the field of corneal studies can reveal opportunities and unexplored areas for future investigation. Comparing the volume of research from Mexico to that of major research contributors in America, Europe, and Asia can also provide insight into the number of ongoing projects in the country.

Mexican research output (basic and clinical) has surged in the last four decades, as depicted in Figure 1. Before 1995, only one or two articles were typically published in indexed journals or books. However, the number of published papers experienced a considerable increase after that period, peaking at 86 in 2021. It is plausible that before that date, research was primarily published by local journals or book editors lacking indexation in international databases, leading to an underreporting of the actual volume of research. This trend underscores the significance of international publications for the recognition and dissemination of new knowledge.

Collaboration among Mexican authors has been extensive, as illustrated in Figure 5. Nevertheless, collaboration with international researchers and other countries remains limited, as evidenced by Figure 4. These findings may serve as a catalyst for fostering increased international cooperation in future research endeavors.

Our study does have limitations. Given that the search methodology aimed to encompass as much corneal and ocular surface research as possible, it was challenging to evaluate the impact of these publications on specific diseases, pathologies, or surgical techniques. Nonetheless, we prioritized ensuring that our study remained as inclusive as possible when determining the influence of a particular country on a specific area of research.

## Conclusions

Although Mexican contributions to corneal and ocular surfaces have increased significantly, they remain marginal at the global level. Interaction with foreign researchers on this topic remains poor. We have identified the main contributors to the field, and our paper contributes to raising awareness of current collaborations. The mechanisms required to boost our scientific output are complex, and identifying and addressing them is a national health priority that will lead to better medical care and education. Moreover, this will potentially result in economic returns that will compensate for our investments. Future studies should address whether this trend of Mexican research staying at the local level continues, as globalization should help researchers connect and expand knowledge.

## References

[1] Hefler L, Tempfer C, Kainz C (1999). Geography of biomedical publications in the European Union, 1990-98. Lancet.

[2] Nichols JJ, Morgan PB, Jones LW, Efron N (2021). 21st century bibliometric analysis of the field of dry eye disease. Clin Exp Optom.

[3] Rubio C, Luna R, Ibarra-Velasco M, Lee Á (2021). Epilepsy: a bibliometric Analysis (1968-2020) of the Instituto Nacional de Neurología y Neurocirugía “Manuel Velasco Suarez” in Mexico. Epilepsy Behav.

[4] Diéguez-Campa CE, Pérez-Neri I, Reyes-Terán G (2021). The 2020 research pandemic: A bibliometric analysis of publications on COVID-19 and their scientific impact during the first months. Arch Cardiol Mex.

[5] Donthu N, Kumar S, Mukherjee D, Pandey N, Lim WM (2021). How to conduct a bibliometric analysis: an overview and guidelines. J Bus Res.

[6] Aria M, Cuccurullo C (2017). bibliometrix: an R-tool for comprehensive science mapping analysis. J Informetr.

[7] Elango B, Rajendran P (2012). Authorship trends and collaboration pattern in the marine sciences literature: a scientometric study. Int J Inf Dissem Technol.

[8] Ramos-Casals M, Brito-Zerón P, Bombardieri S (2020). EULAR recommendations for the management of Sjögren's syndrome with topical and systemic therapies. Ann Rheum Dis.

[9] Chayet AS, Assil KK, Montes M, Espinosa-Lagana M, Castellanos A, Tsioulias G (1998). Regression and its mechanisms after laser in situ keratomileusis in moderate and high myopia. Ophthalmology.

[10] Caceres-Rios H, Tamayo-Sanchez L, Duran-Mckinster C, de la Luz Orozco M, Ruiz-Maldonado R (1996). Keratitis, ichthyosis, and deafness (KID syndrome): review of the literature and proposal of a new terminology. Pediatr Dermatol.

[11] Prospero Ponce CM, Rocha KM, Smith SD, Krueger RR (2009). Central and peripheral corneal thickness measured with optical coherence tomography, Scheimpflug imaging, and ultrasound pachymetry in normal, keratoconus-suspect, and post-laser in situ keratomileusis eyes. J Cataract Refract Surg.

[12] Rabinowitz YS (1998). Keratoconus. Surv Ophthalmol.

[13] Kauffmann T, Bodanowitz S, Hesse L, Kroll P (1996). Corneal reinnervation after photorefractive keratectomy and laser in situ keratomileusis: an in vivo study with a confocal videomicroscope. Ger J Ophthalmol.

[14] Ramírez M, Hernández-Quintela E, Sánchez-Huerta V, Naranjo-Tackman R (2006). Confocal microscopy of corneal flap microfolds after LASIK. J Refract Surg.

[15] Hartl FU, Hayer-Hartl M (2002). Molecular chaperones in the cytosol: from nascent chain to folded protein. Science.

[16] Ragghianti CP, Martínez R, Martins J, Gallo JE (2006). Comparative study of scientific publications in Ophthalmology and Visual Sciences in Argentina, Brazil, Chile, Paraguay and Uruguay (1995-2004). Arq Bras Oftalmol.

[17] Ventura AG, Ventura AJ, Santos SA (2008). Evolutive characteristics of the scientific articles published in the "Arquivos Brasileiros de Oftalmologia" between 1986 and the year 2000 (Article in Portuguese). Arq Bras Oftalmol.

[18] Galván LC, Ríos N, Lansingh VC, Lee Á, Wu L, Lopez E (2018). Analysis of ophthalmological and vision-related publications in Latin America. Arq Bras Oftalmol.

[19] Muccioli C, Campos M, Goldchmit M, Dantas PE, Bechara SJ, Costa VP (2006). Articles in English in the Brazilian archives of ophthalmology: a result of globalization (Article in Portuguese). Arq Bras Oftalmol.

[20] Lira RP, Vieira RM, Gonçalves FA, Ferreira MC, Maziero D, Passos TH, Arieta CE (2013). Influence of English language in the number of citations of articles published in Brazilian journals of ophthalmology. Arq Bras Oftalmol.

[21] Pacheco Aispuro G, Rojas Jácome IB, Martínez Zamora CA (2023). Bibliometric analysis: six decades of Scientific production from a nationwide Institution: Instituto de Seguridad y Servicios Sociales de los Trabajadores del Estado (ISSSTE) from Mexico. Healthcare (Basel).

[22] Milia MF, Brambila CG, Lee Á, Ponce JI (2023). The transformation of medical research in Mexico: a structural analysis of thematic domains, institutional affiliations, authors’ cohorts, and possible correlations. Quant Sci Stud.

[23] Costagliola C, Batterbury M, Dua HS, Mastropasqua L (2014). Latest treatment option and technology advancement in corneal and ocular surface disease. Biomed Res Int.

[24] Sarkar S, Panikker P, D'Souza S, Shetty R, Mohan RR, Ghosh A (2023). Corneal regeneration using gene therapy approaches. Cells.

[25] Hashemi H, Fotouhi A, Yekta A, Pakzad R, Ostadimoghaddam H, Khabazkhoob M (2018). Global and regional estimates of prevalence of refractive errors: systematic review and meta-analysis. J Curr Ophthalmol.

